# Physician Practice Affiliation Drives Site of Care Cost Differentials: An Opportunity to Reduce Healthcare Expenditures

**DOI:** 10.3390/jmahp13030036

**Published:** 2025-07-24

**Authors:** Deepak A. Kapoor, Mark Camel, David Eagle, Lauren C. Makhoul, Justin Maroney, Zhou Yang, Paul Berggreen

**Affiliations:** 1 The Icahn School of Medicine at Mount Sinai, New York, NY 10029, USA; 2 Orthopaedic & Neurosurgery Specialists, Greenwich, CT 06831, USA; camel@onsmd.com; 3 New York Cancer & Blood Specialists, Patchogue, NY 11772, USA; deagle@nycancer.com; 4 Avalere Health, Richmond, VA 23235, USA; lmakhoul@avalere.com; 5 Capital Cardiology Associates, Albany, NY 12211, USA; jmaroney@capitalcardiology.com; 6 Avalere Health, Washington, DC 20005, USA; zhou.yang@avalerehealth.com; 7 Arizona Digestive Health, Phoenix, AZ 85037, USA; paul.berggreen@gialliance.com

**Keywords:** ambulatory surgical procedures/economics, costs and cost analysis/economics, delivery of health care/organization and administration, practice patterns, physicians’/economics, health expenditures, hospital–physician relations

## Abstract

The continued migration of physicians from independent practice to affiliation with larger entities has garnered significant scrutiny. These affiliation models include hospitals and health systems, payers and corporate entities, and management services organizations, which may or may not be private equity (PE)-backed. Data on the impact of different physician affiliation models on cost of care is limited. We examined the relationship between provider affiliation model, site of care (SOC), and cost of care for certain high-volume procedures in procedure-intensive specialties for both Medicare and commercial insurance. We found that hospital-affiliated physicians are least likely—and PE-affiliated physicians are most likely—to provide care in lower-cost settings. For both Medicare and commercial insurance, SOC contributes meaningfully to procedure unit price, which is consistently greater in hospital-based settings. These findings suggest that the physician affiliation model and associated SOC cost differentials contribute materially to healthcare expenditures. As the Medicare cost differentials are set by statute and regulations, strategies such as site-neutral payments are needed to mitigate the monetary impact of historical and future physician practice migration.

## 1. Introduction

Regulatory, administrative, and economic pressures continue to push physicians into affiliation with larger organizations that may offer administrative and financial support [1]. As of 2022, between 41% and 52% of physicians were hospital-affiliated [1,2], and 23% were affiliated with corporate entities [2]. Physicians have also affiliated with private equity (PE)-backed management services organizations (MSOs); however, the prevalence of PE affiliation is comparatively small. Recent reports found that providers affiliated with PE-backed MSOs represent less than 4% of the U.S. healthcare provider market by revenue and that PE-affiliated private practices represented just 6% of physicians in five selected specialties in 2022 [3,4]. Several studies have compared cost and quality metrics for physicians with and without hospital affiliations [5,6,7,8,9], but PE-affiliated models have received attention disproportionate to their prevalence [10,11]. To date, comparative studies between affiliation models have not been performed.

Studies have examined cost-to-charge ratios, net income, and professional fee differentials in affiliated versus unaffiliated physicians; typically, these studies found that provider consolidation was associated with increased healthcare expenditures without concomitant increases in quality [7,9,10,12,13,14]. Similarly, studies comparing costs at different outpatient sites of care (SOCs) show that services are more expensive in hospital-based settings [12,15,16,17,18,19,20].

Reimbursement for healthcare services that differs by SOC is intrinsic to the underlying methodology used to set payment rates. For services performed in the physician office, the Physician Fee Schedule (PFS) sets Medicare professional fees. This uses physician work, practice expense, and malpractice relative value units (RVUs) with a geographic adjustment to calculate the total weight for each service. This weight is then multiplied by a standard national conversion factor to derive the payment amount for each service; many commercial payers and Medicare Advantage (MA) plans also use this model to determine reimbursement. For services performed in facilities (which include hospital outpatient departments (HOPDs) and ambulatory surgery centers (ASCs)), physician work and malpractice RVUs are the same as in the office setting; however, practice expense RVUs are replaced by a “facility fee”. This facility fee is typically substantially higher than the payment associated with practice expense RVUs, and since the professional and work components are unchanged, the aggregate cost to the system and patient for services performed in a facility setting is typically higher than in the office. In addition, facility fees (which are governed by the separate Outpatient Prospective Payment System (OPPS)) are further bifurcated between HOPDs and ASCs.

Importantly, cost changes differ by SOC due to regulations and statute. Changes in office fees under the PFS are subject to statutory budget neutrality requirements, but facility payments are not. As a result, growth in facility fees has outpaced that of professional fees over the last 20 years: 60% (2.4% year-over-year) versus 11% (0.5% year-over-year), respectively, from 2001 through 2021 [21,22]. Furthermore, since the implementation of the Affordable Care Act [23], reimbursements to ASCs are approximately 42% lower than payments to HOPDs for performing the same service [24]. While the impact of SOC on cost has been studied [7,9,12,13,15,17,19], the complex interplay of physician affiliation, SOC trends, and cost has not been thoroughly examined.

We sought to examine the relationship between physician practice affiliation and the relative likelihood and cost of service provision in higher-cost versus lower-cost SOCs across the full spectrum of affiliation models via two questions:

(1) How does practice affiliation model influence SOC dynamics for common procedures in certain specialties?
(2) For Medicare and commercial insurance, how does total service cost vary by SOC across affiliation models?

We studied four physician practice affiliation models—unaffiliated private practice (UPP), PE-affiliated private practice (PEAPP), corporate, and hospital—in cardiology, gastroenterology, orthopedics, and urology. These specialties were chosen using several criteria: (1) the ability to observe meaningful utilization in the Medicare population; (2) PE interest in investment via MSO affiliation; and (3) the ability to assess SOC dynamics for high-cost, high-volume services—specifically, procedures in surgical and imaging-intensive specialties.

## 2. Materials and Methods

We studied four physician specialties, focusing on high-volume services using Medicare claims, payment rates from the Medicare professional and facility fee schedules, and aggregated, de-identified summary information about commercial reimbursement provided by FAIR Health based on claims in the FH NPIC^®^ (National Private Insurance Claims) database.

### 2.1. Physician Specialty Selection and Affiliation Model Assignation

We used multiple data sources to define the physician practice affiliation models included in the study. This included analyzing the existing literature for use of similar methods by affiliation, specialty, and year and comparing the sample size of physicians in each category. The sample size of this study is consistent with previous studies, suggesting the reasonableness of the affiliation methodology utilized [10].

The definitions of UPP, corporate, and hospital correspond to the owner type variable categories of independent, corporate-owned practice, and integrated health-system owned practice, respectively, in the IQVIA OneKey dataset [25]. The OneKey dataset includes frequently updated information on health systems, physicians, advanced practice clinicians, hospitals, and nursing homes nationwide. It contains system- and facility-level data on staffing, beds, and facility type, as well as physician-level data on specialty and affiliations. Data—including the data elements used for this analysis—are collected through a combination of telephone surveys and administrative sources. These data describe relationships between providers and hospitals or group practices; they also describe purchasing and contracting relationships between facilities [25].

With respect to affiliation, IQVIA designates physicians who do not have a parent organization as independent, which this analysis calls UPP. Then, IQVIA designates those with parent organizations as either “hospital” or “corporate”; this analysis refers to the former group as “hospital” or “hospital-affiliated”. Physicians with a “corporate” parent organization designation in the OneKey dataset include physicians in various arrangements, including PEAPP; therefore, we took additional steps to identify the PEAPP cohort in our study.

In order to carve out PEAPP—which we defined as affiliation with a PE-backed MSO, regardless of the PE sponsor’s ownership stake in the MSO—from IQVIA’s corporate category, we obtained the National Provider Identifiers (NPIs) of all physicians within the corporate category who submitted claims to Medicare. By reviewing PitchBook (a private capital markets database), press releases, investor websites, and publicly available lists of PE portfolio companies, we identified PEAPP practices. Next, we matched physician NPIs with the legal business names of PEAPP practices using LexisNexis data, thereby identifying the physicians who belong in the PEAPP category.

Medicare beneficiaries were attributed separately for each specialty to an affiliation model based on an analysis of the 100% Medicare fee-for-service (FFS) data. For the SOC analysis, beneficiaries were attributed to the model where they incurred the highest number of claims, provided they had at least two claims. In cases of a tie involving PEAPP (0.3% or less of the sample across specialties), the beneficiary was assigned to PEAPP to ensure adequate sample size. For ties not involving PEAPP (1.0% or less of the sample across specialties), the beneficiary was assigned randomly to one of the models (Supplemental Table S1). To be included, beneficiaries had to have been continuously enrolled in Medicare Parts A and B throughout the period of the analysis. Please refer to Table S4 in the Supplementary Material for the distribution of physicians and Medicare beneficiaries.

### 2.2. Specialty-Specific Service Selection

FAIR Health commercial data from 2022 were used to select services from each specialty for analysis. Consistent with the literature and to enable direct comparison of the total reimbursement of the same services by SOC, we focused on the procedures that could be—and are consistently—safely and effectively performed across multiple outpatient settings, thereby not considering the inpatient setting and associated costs. To ensure our ability to use a consistent code set across the two analyses, we used commercial data for service code selection, as opposed to Medicare data, due to the more limited availability of commercial claims data than the 100% availability of the Medicare data.

To identify specific procedures for inclusion in the study, we identified the top 15 Current Procedural Terminology^®^ (CPT) codes by contribution to either total payment or total volume for each specialty, with “total” defined as all services billed annually by all providers with the corresponding specialty taxonomy included in the FAIR Health data set. Evaluation and management (E&M) services were excluded due to (1) lack of specialty specificity, (2) disparities in the procedure codes used across SOCs and payer types [26], (3) changes to site-neutral payment policies during the study period [27], and (4) an established body of evidence addressing SOC-related pricing dynamics for E&M services [28,29]. Drug-related services (J codes) were excluded due to (1) challenges calculating reimbursement because units billed on a claim may vary considerably across patients and visits and (2) the inability to distill the influence of drug payment dynamics, such as buy-and-bill and 340B pricing, on acquisition cost.

Once the top codes for each specialty were identified, and exclusions were applied, we narrowed eligibility to codes consistently performed across multiple outpatient SOCs. This was determined based on the distribution of total claims volume across all SOCs; specifically, codes were only included in the study if they had both (1) at least 10% volume in the HOPD and (2) at least 10% volume in one or more lower-cost SOCs (defined as the ASC or the office). Once the volume threshold was met and the available reimbursement data were confirmed to be sufficient, we identified the lower-cost setting for each code (a total of 32 across the four specialties) with the larger portion of total volume for comparison to the HOPD. The result is a comparison of the highest-cost outpatient setting (the HOPD) to the most-utilized lower-cost outpatient setting (either the ASC or the office) for each code. Given the heavy representation of surgical codes in the analyzed set, most services (23 out of 32) utilized the ASC as the comparison SOC, while nine services utilized the office setting.

These selection criteria resulted in a set of services that represent a material portion of each specialty’s revenue and volume in the Medicare and commercial populations. Across all the codes selected, the two compared settings combined represented between 56% and 100% of all commercial volume in 2022. The lower-cost setting (either the ASC or the office, depending on the service) represented between 10% and 84% of total volume, while the HOPD setting represented between 13% and 73% of total volume for each code. For the nine codes for which the office setting was used as the lower cost option, the proportion of volume in the office setting was 35% to 83%, with between 0% and 26% of volume in the ASC setting, indicating sufficient volume for comparison. See Supplemental Table S3E for a complete list of the 32 codes analyzed and the proportion of total commercial claims volume performed in each of the analyzed settings in 2022.

### 2.3. Likelihood of Service Provision and Reimbursement Differential by Site of Care and Affiliation Model

Using SOC as billed on claims, we segmented claims for higher-cost versus lower-cost SOCs. For the outcome variable, we used multivariate logistic regression models, using SAS Enterprise 7.1 software to predict the probability of each service being performed in the higher-cost versus the lower-cost SOC. We modeled each service individually and in aggregate by specialty, from which we calculated the risk-adjusted probability of service use in lower-cost SOCs. The dependent variable is a dummy variable where 1 indicates that the patient received care at a lower cost SOC, which results in the combined volume of patients receiving care at ASC or physician office, and 0 indicates the patient received care at a HOPD. All models were risk-adjusted to control for beneficiary and market-level characteristics. In addition to demographics (age, gender, race), we included Medicare entitlement code (e.g., disability and/or end-stage renal disease) and Medicaid dual enrollment status. To account for selection bias, whereby a sicker Medicare beneficiary is more likely to seek care from a hospital-based [30], we controlled for the patients’ health status by using the Centers for Medicare and Medicaid Services (CMS) Hierarchical Condition Category (HCC) risk score and death within the year. Furthermore, we controlled for social determinants of health using ZIP code-level median household income and rural versus urban location, and we controlled for the local market structure using the predominant affiliation model in the county.

Based on the regression results, we calculated the risk-adjusted predicted probability of beneficiaries receiving care in the higher- versus lower-cost SOC, by affiliation model, for the selected procedures in each specialty. We also weighed the overall probability by the share of the volume of beneficiaries within each specialty to demonstrate the average probability of receiving care in the lower-cost SOC across the population. The risk adjustment was conducted by applying the same beneficiary- and county-level characteristics as previously described to beneficiaries attributed to each affiliation model.

To identify differences in reimbursement by SOC in the commercial market, we obtained summarized statistics for 2022 commercial claims data. FAIR Health provided median national allowed amounts for the selected services using claim-level data reflecting the contracted negotiated rate between providers and payers, including deductible and co-insurance. Commercial reimbursement was analyzed by SOC, fee type, and procedure; we did not consider affiliation model, as those data were unavailable. For the Medicare reimbursement analysis, we used the 2024 PFS to identify facility and non-facility professional fees, as well as the 2024 OPPS and ASC Fee Schedules to identify facility fees. For HOPD and ASC rates for the Medicare and commercial analyses, the facility fee was combined with the professional fee to calculate total reimbursement.

## 3. Results

### 3.1. Service-Specific Differences in Reimbursement and Site of Care

**Figure 1:**
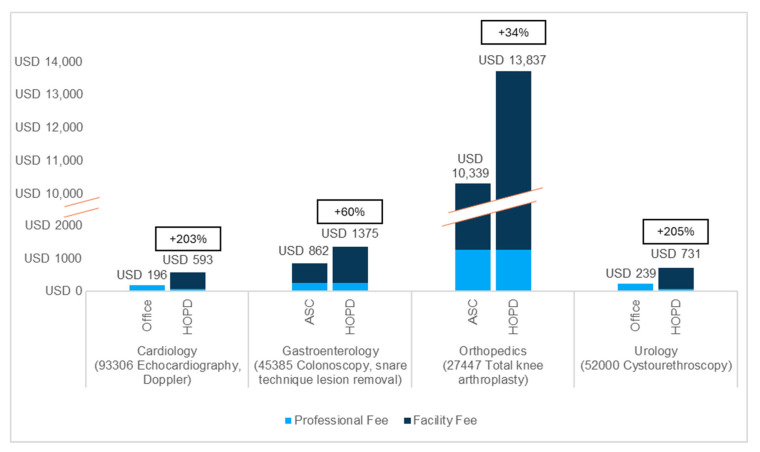
Reimbursement for HOPD vs. lowest-cost site of care (ASC or office) for highest-volume selected procedure per specialty, Medicare (2024). Total payment (combined professional and facility fees) and the percentage additional cost in the HOPD setting are labeled at the top of each bar. We opted to use the most recent year of available rate data, as we do not expect meaningful differences in payment levels between 2022 (the most recent full year of utilization data available) and 2024.

**Figure 2:**
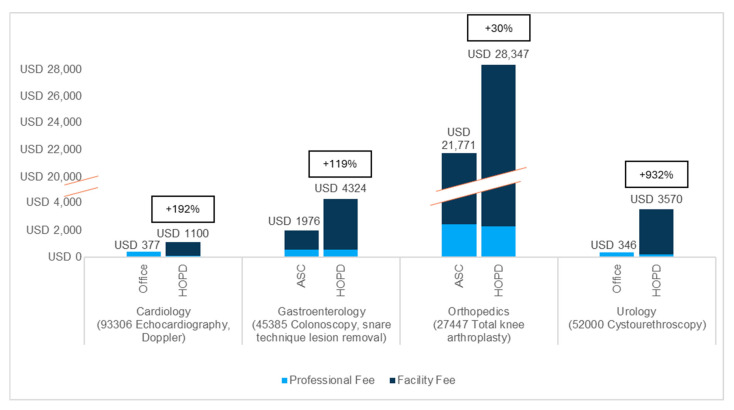
Reimbursement for HOPD vs. lowest-cost site of care (ASC or office) for highest-volume selected procedure per specialty, commercial (2022). Total payment (combined professional and facility fees) and the percentage additional cost in the HOPD setting are labeled at the top of each bar.

Figure 1 and Figure 2 summarize the relative differences in reimbursement by SOC for the most common codes assessed—by volume, per specialty—for Medicare (Figure 1) and commercial insurance (Figure 2) for the single lowest site of service for which at least 10% of services were performed. In all cases, no SOC, other than the office, ASC, or HOPD, met the 10% threshold to be included in the analysis. For all codes analyzed, the HOPD was the highest-cost outpatient SOC for Medicare and commercial insurance, while the office was the lowest-cost SOC for urology and cardiology and the ASC the lowest-cost SOC for gastroenterology and orthopedics. When considered in aggregate, total reimbursement for the higher-cost SOC was 124% to 861% and 111% to 1346% of the combined reimbursement for the lower-cost SOCs (ASC and office) for Medicare and commercial insurance, respectively. All code-level data are available in Supplemental Table S3A–D.

For both higher-cost and lower-cost SOCs, commercial reimbursement is higher than Medicare reimbursement across all codes analyzed (Supplemental Table S3C). Further, the cost difference in dollars between the higher-cost and lower-cost SOC is greater in commercial insurance than in Medicare for all but one of the analyzed codes (CPT 22551, anterior interbody arthrodesis of the cervical spine).

**Figure 3:**
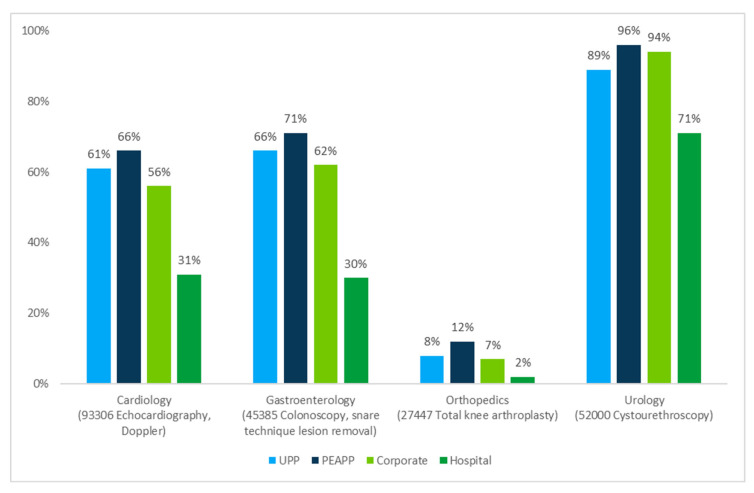
Risk-adjusted probability of highest volume selected procedure per specialty being provided in lower-cost settings (ASC or office, combined), by affiliation model, Medicare (2022).

For each specialty’s most common code by volume, Figure 3 shows differences in the probability of service provision in either of the lower-cost SOCs (ASC or office combined) across models. The results demonstrate the higher probability of receiving care in lower-cost SOCs for beneficiaries treated by PEAPP physicians and the lower probability of receiving care in lower-cost SOCs for beneficiaries treated by hospital-affiliated physicians (statistically significant (*p* < 0.01) across all codes). In any model, PEAPP physicians were most likely to provide twenty-nine of the thirty-two selected specialty services (including four services that tied for most likely) in lower-cost SOCs, from 2% for CPT code 23472 (arthroplasty, glenohumeral joint) to 96% for CPT code 52000 (cystourethroscopy). Further details from our results are in Supplemental Table S3D; please refer to Supplemental Table S5 for the regression results and Supplemental Table S7 for the 95% confidence interval.

### 3.2. Specialty-Level Differences in Specific Service Provision by Site of Care Across Affiliation Models

**Figure 4:**
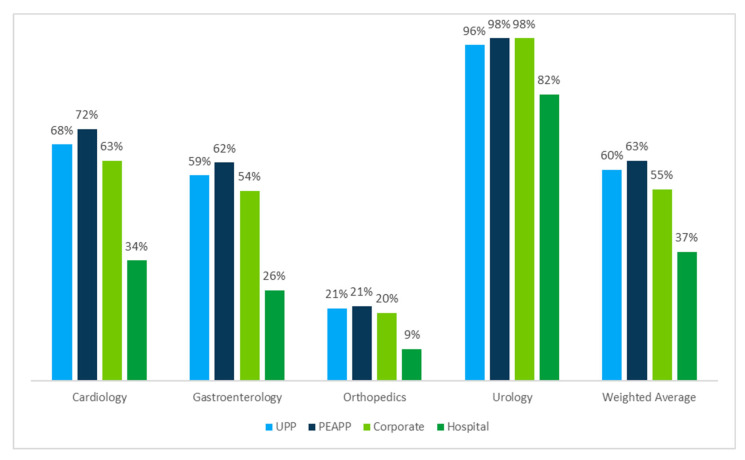
Risk-adjusted probability of selected specialty-specific services being provided in lower-cost settings (ASC or office, combined), by specialty and affiliation model, Medicare (2022).

Observed trends in lower-cost SOC use for individual services persist across affiliation models when aggregated at the specialty level. When weighted across specialties, beneficiaries attributed to PEAPP physicians had the highest average risk-adjusted probability of receiving specialty-specific services in a lower-cost SOC (63%) (Figure 4). The weighted average risk-adjusted probability across specialties was 60% for beneficiaries attributed to UPP physicians and 55% for beneficiaries attributed to corporate physicians. Beneficiaries attributed to hospital-affiliated physicians had the lowest weighted average risk-adjusted probability of receiving specialty-specific services in lower-cost SOCs (37%). The differences in practice patterns are clearest in cardiology and gastroenterology, where beneficiaries attributed to PEAPP physicians are more than twice as likely to receive specialty-specific services in lower-cost settings than those attributed to hospital-based physicians (72% vs. 34% (*p* < 0.01) and 62% vs. 26% (*p* < 0.01), respectively), even after risk adjustment. The difference in risk-adjusted probability between the PEAPP and hospital models is statistically significant (*p* < 0.01) across all specialties and codes assessed. Results of individual code analyses are in Supplemental Table S3D; please refer to Supplemental Table S6 for the regression results and Supplemental Table S7 for the 95% confidence interval.

## 4. Discussion

This study offers a perspective on the likelihood that a beneficiary receives select specialty procedures in lower-cost SOCs based on their physician’s affiliation model. We found that: (a) beneficiaries of PEAPP physicians are most likely (and beneficiaries of hospital-affiliated physicians are least likely) to receive a specialty-specific procedure in a lower-cost setting; (b) the facility fee is the largest driver of the difference in procedure cost across settings; and (c) these differences are larger for commercial insurance than for Medicare.

These findings are especially interesting when considered alongside the ubiquity and magnitude of additional costs attributable to the HOPD when an alternative, lower-cost SOC exists. For example, for CPT code 93306 (transthoracic echocardiogram), the total Medicare reimbursement in the HOPD is 303% of the total reimbursement in the office (USD 593 vs. USD 196), primarily due to a USD 526 facility fee in the HOPD. The impact on total cost is greater in commercial insurance, where the HOPD rate (USD 1100, including a USD 995 facility fee) is 292% of the office rate (USD 377). Even for a service—for example, CPT 45385 (colonoscopy, snare technique lesion removal)—for which the lower-cost SOC (i.e., the ASC) also includes a facility fee, the total HOPD reimbursement is 160% of the ASC rate in Medicare (a difference of USD 513) and 219% in commercial insurance (a difference of USD 2348).

We recognize that the relevance of this analysis would be limited if SOC patterns were stable or indeed reverted to inpatient services. While we acknowledge this concern, analysis of actual utilization trends illustrates that, from 2000 to 2023, other than an aberrant decrease in HOPD services during the COVID-19 pandemic, there was a 19% decrease in inpatient admissions per one thousand individuals with a simultaneous 31% increase in HOPD visits [31]. Given the unabated nature of this trend, we believe that this analysis, which reviews an understudied impact on cost of care, that of physician SOC migration, is both timely and relevant.

Our findings illustrate why SOC dynamics are a significant driver of the impact of physician affiliation on cost. Of the twenty-two codes we evaluated where the ASC is the lower-cost SOC, thirteen (59%) had a higher commercial professional fee in the ASC than in the HOPD. Taken alone, this finding would erroneously suggest that beneficiaries treated by physicians who are more likely to use the ASC setting—such as PEAPP physicians—usually receive more expensive care. However, for all thirteen of these services, the incremental facility fee attributable to the HOPD far exceeds the incremental professional fee in the ASC (Supplemental Table S3B). Such cases show that, even if physician pay increases in lower-cost settings via increased professional fees, such an increase is outweighed by the increased healthcare expenditure attributable to the facility fee in higher-cost settings.

This trend shows how physician migration can create new costs via at least two mechanisms. First, when a hospital acquires a physician practice, certain services may be entitled to a HOPD facility fee simply because of the change in the billing entity. Second, hospital systems increasingly view ancillary services as revenue drivers [32], and employed physicians are strongly encouraged to use hospital-owned facilities. Indeed, data suggest that hospitals generate approximately USD 2.4 million in profit per employed physician [33]. These actions increase costs without any change in care or its delivery [34,35,36].

Payments under the Medicare system are non-negotiable and determined by the OPPS and PFS, but fees paid by commercial insurance can be negotiated. Given their volume, hospitals have greater leverage than physicians or ASCs in negotiating commercial rates, potentially further exacerbating SOC cost differentials. Considering prior studies indicating that the use of higher-cost SOCs for services like cardiac imaging and colonoscopies is not fully explained by differences in patient acuity [15], the varying proclivity of physicians by affiliation model to use a higher-cost SOC when a lower-cost SOC is available is notable, especially given the risk-adjusted nature of our findings.

Importantly, these findings are not limited to the specialties and services in our analysis. Other thorough studies, including by the American Medical Association and the Medicare Payment Advisory Commission (MedPAC), indicate the persistence of these phenomena across numerous specialties and service categories, including primary care, diagnostic radiology, obstetrics/gynecology, surgery, psychiatry, neurology, dermatology, otolaryngology, oncology, drug administration, and diagnostics [15,37,38,39,40]. Indeed, a comparison of the 2025 HOPD and ASC fee schedules reveals that, for 100% of the 3692 HCPCS codes payable at both SOCs across all specialties, payment is higher in the HOPD versus the ASC [41].

Despite the comprehensive nature of the analysis, this study has limitations. First, the detailed manual review required to distinguish PEAPP from corporate affiliation may not have identified every PEAPP practice, and the use of OneKey’s secondary data may include some sampling error. This limitation was mitigated, as the OneKey data is collected and constructed by multiple data sources to identify the relationships between the healthcare personnel and healthcare organizations. The data construction includes primary data collection from healthcare organizations, external industry sources, transactional data, direct client submissions, and direct data validation. This construct—one of a modern data science project—is unique from the traditional single-source data curation given the overall challenges in identifying ownership of healthcare entities. We felt that use of this source was appropriate due to its consistent use across similar published evidence [3,42,43,44]. Second, this study considered only a subset of specialty-specific procedures, but the inclusion criteria—high thresholds for contribution to total payment and volume—ensured that the analyzed codes were sufficiently representative. An additional limitation was random assignation of beneficiaries to affiliation models when ties in claim counts occurred; however, this affected less than 1% of the total beneficiary sample. Although these multivariate analyses could not be replicated for the commercial population due to the unavailability of underlying claims data, the greater magnitude of commercial reimbursement in the HOPD versus the ASC and office SOCs suggests meaningful opportunity to reduce healthcare expenditure through SOC optimization.

We also acknowledge that, due to data limitation, we cannot identify the case mix by facility or by individual practices; further, to keep the analysis consistent with one set of beneficiary attribution criteria, we did not control for facility-level case mix variations. This may be seen as a limitation of the study, as a physician’s case mix can be associated with SOC, in that it may shape a physician’s choice of setting for a particular service depending on the clinical appropriateness for a given patient or patient population. In fact, some researchers have suggested that SOC cost differentials are the result of patient demographic issues, including lower socioeconomic status and greater incidence of patient co-morbidities [30]. We acknowledge these findings and agree that (1) the nuance of patients seeking different types of procedures is highly diversified, and (2) the complexity of the comorbidities and general health at the patient level strongly correlates with the site of care of the procedure. And while we agree that those patients with more complex conditions are more likely to warrant care from a hospital than other outpatient care settings, we offer three reasons why we do not believe case mix is a limitation of our study.

First, our analysis does adjust for patient risk, and we believe our methodology, which includes not only HCC but also death year, demographics, and zip code level Social Determinants of Health to predict the probability to seek care at high vs. low-cost setting, adequately compensates for any increased comorbidities in the HOPD cohort as described in the manuscript. Second, this question has been extensively researched by MedPAC, whose 2023 report to Congress found that, for most procedures, the differential in risk profiles of patients treated across SOCs has an “insignificant effect on hospital charges”, concluding that, “adjustments for patient severity are not necessary for the services in the 66 APCs in our analysis” [39]. Finally, our analysis excludes the inpatient setting (which, given its variable payment structure and acute level of care is not a relevant comparison to outpatient procedure expenditures). Given the extensive work previously done addressing the limited impact of patient case mix and cost, as well as our efforts to normalize these parameters across SOCs, we believe that any impact to our findings that may possibly result from case mix differentials would be nominal and not affect our conclusions.

One could view the inherent utilization constraints on providers in various SOCs as a limitation of this analysis. While we acknowledge this perspective, we feel that this very constraint makes the case for payment reform. Policies that limit physicians’ choice of affiliation model could exacerbate the healthcare expenditure crisis if models that prioritize lower-cost SOCs are constrained, not to mention the perceived potential loss of physician autonomy, which has been identified as a significant cause of burnout [45]. Stakeholders should strongly support the rights of individual practitioners to choose the site of care that best suits their capabilities and lifestyle choices. While we can and should respect providers’ choice of workplace, we cannot lose sight of the downstream impact of that choice. Fundamentally, the question is not whether the physician should be incentivized or penalized for their choice of SOC, but whether any individual patient or the healthcare system should bear the cost burden of that choice. There is no fundamentally fairer principle than equal pay for equal work—indeed, it is at the core of the relative value system around which all payments are made. For a multitude of reasons, new graduates are increasingly seeking employment over independent practice, and independent practitioners are migrating toward employment as well [1]. It is likely that the trend of UPP physicians migrating to—and new physician graduates choosing—employment over independent practice will continue. Eliminating payment differential, thereby linking payment to the service itself and not the site at which the service is performed, would effectively eliminate any impact on reimbursement that physicians may incur due to potential referral constraints across sites of service.

As the trend toward affiliation seems inexorable, stakeholders have an interest in minimizing the cost associated with different affiliation models, which this study has shown to be variable. A more reasoned approach that would not constrain provider rights to choose their preferential practice SOC would be to acknowledge that physician affiliation trends are changing and to create a level playing field such that the choice of affiliation does not impact cost of care. As Medicare SOC payment differentials are set by statute and regulations, policymaker interest in site-neutral payment policies continues to grow. MedPAC [39], the U.S. Department of Health and Human Services Office of Inspector General [46], and the U.S. Government Accountability Office [47] all recently conducted analyses assessing SOC payment differences given their impact on Medicare expenditures. Additionally, Congress introduced several pieces of legislation, beginning in 2023, to enact various reforms related to SOC payment differentials, targeting areas including drug administration services and removing exemptions for HOPDs. In fact, the House of Representatives passed the Lower Costs, More Transparency Act [48], which included a policy to equalize Medicare payments for the administration of drugs, by a vote of 320-71. Congress continues to focus on policies that establish site-neutral payment across settings by reducing the inpatient and HOPD Medicare payment rates for procedures such as X-rays, colonoscopies, cancer screenings, heart-imaging procedures (e.g., echocardiograms and nuclear cardiology), and follow-up appointments. Such policies could disincentivize the acquisition of unaffiliated practices that operate independent outpatient clinics, surgery centers, and physician offices by large hospital systems that, post-acquisition, simply offer the same procedures at higher Medicare payment rates. This strategy would have the positive dual impact of slowing the acquisition of UPP groups and generating significant savings for Medicare. Thus far, successful efforts to implement these policies have been limited, as large, affiliated hospital groups understandably push to maintain the status quo to their benefit. Payment differentials persist for healthcare services in Medicare, as well as—and often to a greater extent—in commercial insurance. Efforts have been made regionally to address commercial market SOC differentials via state policy; however, data suggest that site-neutral payment could save as much as USD 898 billion in the commercial market over the next decade [49,50,51,52]. To date, the discussion of SOC-related payment differentials has not adequately considered trends in physician practice affiliation, and such conversations are overdue.

## 5. Conclusions

This study demonstrates that physician affiliation model has a significant impact on the likelihood that patients will receive select specialty services in lower-cost SOCs. Beneficiaries cared for by PEAPP physicians are most likely across all affiliation models—including UPP—to receive specialty care at lower-cost SOCs, while those affiliated with hospital-based physicians are most likely to receive care at higher-cost SOCs. As most physician affiliation occurs via hospital employment, it is likely that, without action, costs associated with this phenomenon will continue to rise. Our findings suggest that the cost impact of migration in physician affiliation may be mitigated by payment strategies that reduce SOC payment differentials, and federal policymakers have the power to change the payment structure to better align Medicare payment rates with patient needs, access to care, and cost savings. Hospitals, however, continue to make opposition to these efforts their key legislative priority.

## Data Availability

The Medicare claims data that support the findings of this study are not openly available and were accessed via a research collaboration with Inovalon, Inc. and governed by a research-focused Data Use Agreement with the Centers for Medicare and Medicaid Services. The 2024 Medicare Physician Fee Schedule is available from https://www.govinfo.gov/content/pkg/FR-2023-11-22/pdf/2023-24293.pdf (accessed on 22 November 2023). The commercial insurance data that supports the findings of this study is based, in part, upon aggregated, de-identified, summary data drawn from healthcare claims data compiled and maintained by FAIR Health, Inc. The authors of this article are solely responsible for the research and conclusions reflected in this article. FAIR Health Inc. is not responsible for the conduct of the research or for any of the opinions expressed in this article. The SAS programming code specifying the regression analyses is available upon request. Programming codes to assign physician practice affiliations are not available.

## Supplementary Materials

The following supporting information can be downloaded at https://www.mdpi.com/article/10.3390/jmahp13030036/s1: Table S1: Beneficiary Assignment Involving Ties Across Multiple Affiliation Models, by Specialty (2022); Table S2: Specialty-Specific Service Selection; Table S3A: Reimbursement by Procedure Code, Medicare; Table S3B: Reimbursement by Procedure Code, Commercial; Table S3C: Total Reimbursement by Procedure Code, Medicare vs. Commercial; Table S3D: Summary Statistics by Procedure Code, Medicare; Table S3E: Summary Statistics by Procedure Code, Commercial; Table S4: Summary table of the distribution of physicians by specialty and affiliation; Table S5: Regression Results of Figure 3; Table S6: Regression Results of Figure 4; Table S7: Confidence Intervals of Results Presented in Figure 3 and Figure 4.
