# Supplementary material for: Physician Practice Affiliation Drives Site of Care Cost Differentials: An Opportunity to Reduce Healthcare Expenditures
Source: J Mark Access Health Policy. 2025 Jul 24;13(3):36. doi: 10.3390/jmahp13030036 (PMC12371938; doi:10.3390/jmahp13030036)
Supplement: Supplementary file 1 [file jmahp-13-00036-s001.zip › jmahp-3637728-supplementary.pdf]

Supplementary Information

Data Sources Disclosure

Supplementary Table S1 Beneficiary Assignment Involving Ties Across Multiple Affiliation Models, by Specialty (2022)

Supplementary Table S2 Specialty-Specific Service Selection

Supplementary Table S3A Reimbursement by Procedure Code, Medicare

Supplementary Table S3B Reimbursement by Procedure Code, Commercial

Supplementary Table S3C Total Reimbursement by Procedure Code, Medicare vs. Commercial

Supplementary Table S3D Summary Statistics by Procedure Code, Medicare

Supplementary Table S3E Summary Statistics by Procedure Code, Commercial

Supplementary Table S4 Summary table of the distribution of physicians by specialty and affiliation

Supplementary Table S5 Regression Results of Figure 3

Supplementary Table S6 Regression Results of Figure 4

Supplementary Table S7 Confidence Intervals of Results Presented in Figures 3 and 4

Data Sources Disclosure

Research for this article is based, in part, upon aggregated, de-identified, summary data drawn from healthcare claims data compiled and maintained by FAIR Health, Inc. The authors of this article are solely responsible for the research and conclusions reflected in this article. FAIR Health, Inc. is not responsible for the conduct of the research or for any of the opinions expressed in this article.

Supplementary Table S1 Beneficiary Assignment Involving Ties Across Multiple Affiliation Models, by Specialty (2022)

Specialty	Total Beneficiaries with 2+ Claims in At Least 1 Model	Beneficiaries with Any Ties Across 2 Models		Beneficiaries with Ties Including PEAPP		Beneficiaries with Ties not Including PEAPP	
	(N)	(N)	(%)	(N)	(%)	(N)	(%)
**Cardiology**	6,563,191	72,309	**1.1%**	3,526	**0.1%**	68,783	**1.0%**
**Gastroenterology**	1,792,795	16,077	**0.9%**	4,945	**0.3%**	11,132	**0.6%**
**Orthopedics**	2,977,561	30,250	**1.0%**	4,802	**0.2%**	25,448	**0.9%**
**Urology**	1,960,966	8,815	**0.4%**	2,467	**0.1%**	6,348	**0.3%**

Supplementary Table S2 Specialty-Specific Service Selection

Specialty	Description of Included Codes	# Selected Codes	% Total 2022 Commercial Payments for Specialty	% Total 2022 Commercial Claims (Units) for Specialty	% 2022 Commercial Claims (Units) for Included Codes in Lower Cost Setting (Office or ASC)	% 2022 Commercial Claims (Units) for Included Codes in HOPD
**Cardiology**	• Echocardiography (93306, 93351)<br>• Duplex scan (93880, 93970)<br>• Other: myocardial perfusion imaging (78452)	5	**42.1%**	**17.4%**	**48.3%**	**33.6%**
**Gastroenterology**	• Esophagogastroduodenoscopy (EGD) – i.e., upper endoscopy (43235, 43239, 43248, 43249)<br>• Colonoscopy – diagnostic, biopsy, and surgical (45378, 45380, 45381, 45384, 45385, 45390)	10	**57.9%**	**47.7%**	**53.8%**	**36.1%**
**Orthopedics**	• Arthroplasty – i.e., total joint replacement (23472, 27130, 27447)<br>• Arthroscopy (29826, 29827, 29881)<br>• Other: arthrodesis (22551), ACL repair (29888), laminectomy (63047), neuroplasty of carpal nerve (64721)	10	**26.5%**	**3.5%**	**34.7%**	**54.9%**
**Urology**	• Cystourethroscopy (52000, 52310, 52332, 52356, 52442)<br>• Other: lithotripsy (50590), prostate biopsy (55700)	7	**24.1%**	**9.3%**	**55.7%**	**29.4%**
	**Total Codes**	**32**				

Supplementary Table S3A Reimbursement by Procedure Code, Medicare

		Medicare Total Reimbursement: Professional vs. Facility Fee Breakdown					
**Code**	**Code Description - Patient Language**	**Higher-Cost SOC (HOPD)**			**Lower-Cost SOC (ASC or Office)**		
		**Prof.**	**Facility**	**Total**	**Prof.**	**Facility**	**Total**
**Cardiology**							
78452*	Myocardial SPECT imaging	$74	$1,354	$1,429	$433	$0	$433
93306*	Echocardiography, Doppler	$67	$526	$593	$196	$0	$196

93351*	Echocardiography, stress test	$81	$526	$607	$232	$0	$232
93880*	Extracranial arteries, bilateral study	$37	$234	$271	$190	$0	$190
93970*	Extremity veins, bilateral study	$32	$234	$266	$187	$0	$187
**Gastroenterology**							
43235	Esophagogastroduodenoscopy, diagnostic	$121	$865	$985	$121	$470	$591
43239	Esophagogastroduodenoscopy, biopsy	$136	$865	$1,001	$136	$470	$607
43248	Esophagogastroduodenoscopy, guide wire	$163	$865	$1,028	$163	$470	$634
43249	Esophagogastroduodenoscopy, balloon dilation	$151	$1,815	$1,966	$151	$832	$983
45378	Colonoscopy, diagnostic	$181	$872	$1,053	$181	$474	$656
45380	Colonoscopy, biopsy	$197	$1,126	$1,323	$197	$612	$809
45381	Colonoscopy, submucosal injection(s)	$197	$1,126	$1,322	$197	$612	$809
45384*	Colonoscopy, lesion removal with forceps	$224	$1,126	$1,350	$487	$0	$487
45385	Colonoscopy, snare technique lesion removal	$249	$1,126	$1,375	$249	$612	$862
45390	Colonoscopy, mucosal resection	$326	$2,678	$3,004	$326	$1,349	$1,675
**Orthopedics**							
22551	Arthrodesis, anterior interbody	$1,714	$12,553	$14,267	$1,714	$8,870	$10,584
23472	Arthroplasty, glenohumeral joint	$1,446	$17,775	$19,221	$1,446	$14,003	$15,449
27130	Total hip arthroplasty	$1,286	$12,553	$13,839	$1,286	$9,244	$10,530
27447	Total knee arthroplasty	$1,284	$12,553	$13,837	$1,284	$9,055	$10,339
29826*	Shoulder arthroscopy, subacromial decompression	$170	**	$170	$170	$0	$170
29827	Shoulder arthroscopy, rotator cuff repair	$1,074	$6,823	$7,897	$1,074	$3,393	$4,467
29881	Knee arthroscopy, meniscectomy	$549	$3,087	$3,637	$549	$1,519	$2,068
29888	Arthroscopic ACL repair	$979	$6,823	$7,803	$979	$4,500	$5,479
63047	Laminectomy, facetectomy and foraminotomy	$1,119	$6,823	$7,942	$1,119	$3,393	$4,512
64721	Neuroplasty and/or transposition	$445	$1,842	$2,287	$445	$898	$1,343
**Urology**							
50590	Lithotripsy, extracorporeal	$571	$3,325	$3,896	$571	$1,626	$2,197
	shock wave						
52000*	Cystourethroscopy	$79	$652	$731	$239	$0	$239
52310*	Cystourethroscopy, removal of foreign body	$149	$1,943	$2,092	$319	$0	$319
52332	Cystourethroscopy, insertion of stent	$153	$3,325	$3,478	$153	$1,626	$1,779

52356	Cystourethroscopy, ureteroscopy/pyeloscopy, lithotripsy and stent	$407	$4,935	$5,343	$407	$2,471	$2,879
52442*	Cystourethroscopy, insertion of transprostatic implant	$50	**	$50	$854	$0	$854
55700*	Prostate biopsy	$128	$1,943	$2,071	$241	$0	$241

Notes:

- A facility fee of $0 in the lower-cost setting indicates that the lower-cost SOC for that service is the office; these have been marked with *. For services with a non-zero facility fee in the lower-cost setting, the lower-cost setting is the ASC.
- Professional and facility fees may not always add perfectly to the total reimbursement because of rounding.
- **These codes are add-on codes with no separately payable facility fee.

Supplementary Table S3B Reimbursement by Procedure Code, Commercial

		Commercial Total Reimbursement: Professional vs. Facility Fee Breakdown					
Code	Code Description - Patient Language	Higher-Cost SOC (HOPD)			Lower-Cost SOC (ASC or Office)		
		Prof.	Facility	Total	Prof.	Facility	Total
**Cardiology**							
78452*	Myocardial SPECT imaging	$345	$2240	$2,584	$622	$0	$622
93306*	Echocardiography, Doppler	$105	$995	$1,100	$377	$0	$377
93351*	Echocardiography, stress test	$147	$952	$1,099	$437	$0	$437
93880*	Extracranial arteries, bilateral study	$143	$482	$625	$263	$0	$263
93970*	Extremity veins, bilateral study	$59	$532	$591	$234	$0	$234
**Gastroenterology**							
43235	Esophagogastroduodenoscopy, diagnostic	$230	$3,549	$3,779	$207	$1,008	$1,215
43239	Esophagogastroduodenoscopy, biopsy	$231	$3,617	$3,848	$223	$1,110	$1,333
43248	Esophagogastroduodenoscopy, guide wire	$268	$3,481	$3,749	$244	$981	$1,225
43249	Esophagogastroduodenoscopy, balloon dilation	$244	$4,600	$4,844	$213	$1,724	$1,936
45378	Colonoscopy, diagnostic	$391	$3,011	$3,402	$393	$1,161	$1,554
45380	Colonoscopy, biopsy	$420	$3,757	$4,177	$422	$1,312	$1,734
45381	Colonoscopy, submucosal injection(s)	$169	$4,491	$4,660	$176	$1,363	$1,538
45384*	Colonoscopy, lesion removal with forceps	$393	$3,792	$4,185	$746	$0	$746
45385	Colonoscopy, snare technique lesion removal	$542	$3,782	$4,324	$558	$1,418	$1,976
45390	Colonoscopy, mucosal resection	$674	$5,571	$6,245	$657	$1,993	$2,650
**Orthopedics**							
22551	Arthrodesis, anterior interbody	$2,771	$25,190	$27,961	$2,969	$22,111	$25,080
23472	Arthroplasty, glenohumeral joint	$2,526	$28,592	$31,118	$2,561	$21,661	$24,222
27130	Total hip arthroplasty	$2,415	$26,873	$29,288	$2,436	$20,140	$22,576
27447	Total knee arthroplasty	$2,308	$26,038	$28,347	$2,460	$19,311	$21,771
29826	Shoulder arthroscopy, subacromial decompression	$411	$12,196	$12,607	$489	$6,590	$7,080
29827	Shoulder arthroscopy, rotator cuff repair	$1,793	$16,803	$18,596	$1,796	$8,558	$10,354
29881	Knee arthroscopy, meniscectomy	$878	$7,589	$8,467	$902	$3,495	$4,397
29888	Arthroscopic ACL repair	$1,880	$18,574	$20,454	$1,768	$9,122	$10,889
63047	Laminectomy, facetectomy and foraminotomy	$1,763	$13,916	$15,679	$1,883	$7,733	$9,616

64721	Neuroplasty and/or transposition	$640	$5,695	$6,334	$645	$2,563	$3,207
**Urology**							
50590	Lithotripsy, extracorporeal	$877	$8,968	$9,845	$850	$4,809	$5,659
	shock wave						
52000*	Cystourethroscopy	$217	$3,354	$3,570	$346	$0	$346
52310*	Cystourethroscopy, removal of foreign body	$286	$4,394	$4,680	$468	$0	$468
52332	Cystourethroscopy, insertion of stent	$216	$8,810	$9,026	$173	$3,696	$3,869
52356	Cystourethroscopy, ureteroscopy/pyeloscopy, lithotripsy and stent	$690	$10,317	$11,007	$678	$4,151	$4,829
52442*	Cystourethroscopy, insertion of transprostatic implant	$90	$11,571	$11,661	$1,458	$0	$1,458
55700*	Prostate biopsy	$245	$5,137	$5,382	$400	$0	$400

Notes:

- For each code, the lower-cost SOC is the same in the commercial data as in the Medicare data; those using the office setting as the lower-cost SOC have been marked with *.
- Professional and facility fees may not always add perfectly to the total reimbursement because of rounding.

Supplementary Table S3C Total Reimbursement by Procedure Code, Medicare vs. Commercial

		Total Reimbursement, Medicare vs. Commercial			
**Code**	**Code Description - Patient Language**	**Higher-Cost SOC (HOPD)**		**Lower-Cost SOC (ASC or Office)**	
**Cardiology**		**Commercial Minus Medicare**	**Commercial as a % of Medicare**	**Commercial Minus Medicare**	**Commercial as a % of Medicare**
78452*	Myocardial SPECT imaging	$1,156	181%	$189	144%
93306*	Echocardiography, Doppler	$507	185%	$181	192%
93351*	Echocardiography, stress test	$493	181%	$206	189%
93880*	Extracranial arteries, bilateral study	$354	231%	$73	138%
93970*	Extremity veins, bilateral study	$325	222%	$47	125%
**Gastroenterology**					
43235	Esophagogastroduodenoscopy, diagnostic	$2,793	383%	$624	206%
43239	Esophagogastroduodenoscopy, biopsy	$2,847	384%	$726	220%
43248	Esophagogastroduodenoscopy, guide wire	$2,721	365%	$591	193%
43249	Esophagogastroduodenoscopy, balloon dilation	$2,878	246%	$953	197%
45378	Colonoscopy, diagnostic	$2,349	323%	$899	237%
45380	Colonoscopy, biopsy	$2,854	316%	$925	214%
45381	Colonoscopy, submucosal injection(s)	$3,338	352%	$729	190%
45384*	Colonoscopy, lesion removal with forceps	$2,836	310%	$1,478	404%
45385	Colonoscopy, snare technique lesion removal	$2,949	315%	$1,114	229%
45390	Colonoscopy, mucosal resection	$3,241	208%	$975	158%
**Orthopedics**					
22551	Arthrodesis, anterior interbody	$13,693	196%	$14,495	237%
23472	Arthroplasty, glenohumeral joint	$11,897	162%	$8,773	157%
27130	Total hip arthroplasty	$15,449	212%	$12,046	214%
27447	Total knee arthroplasty	$14,510	205%	$11,432	211%
29826	Shoulder arthroscopy, subacromial decompression	$12,437	**	$6,910	**
29827	Shoulder arthroscopy, rotator cuff repair	$10,699	235%	$5,887	232%
29881	Knee arthroscopy, meniscectomy	$4,830	233%	$2,329	213%
29888	Arthroscopic ACL repair	$12,651	262%	$5,410	199%
63047	Laminectomy, facetectomy and foraminotomy	$7,737	197%	$5,104	213%

64721	Neuroplasty and/or transposition	$4,048	277%	$1,865	239%
**Urology**					
50590	Lithotripsy, extracorporeal	$5,949	253%	$3,462	258%
	shock wave				
52000*	Cystourethroscopy	$2,840	489%	$107	145%
52310*	Cystourethroscopy, removal of foreign body	$2,588	224%	$149	147%
52332	Cystourethroscopy, insertion of stent	$5,548	260%	$2,090	217%
52356	Cystourethroscopy, ureteroscopy/pyeloscopy, lithotripsy and stent	$5,665	206%	$1,950	168%
52442*	Cystourethroscopy, insertion of transprostatic implant	$11,611	**	$605	**
55700*	Prostate biopsy	$3,311	260%	$159	166%

Notes:

- For each code, the lower-cost SOC is the same in the commercial data as in the Medicare data; those using the office setting as the lower-cost SOC have been marked with *.
- **These codes are add-on codes with no separately payable facility fee.

Supplementary Table S3D Summary Statistics by Procedure Code, Medicare

		Probability of Service Provision in Lower-Cost Settings, Medicare***				Cost, HOPD Relative to Lower-Cost Setting (ASC or Office) Above 10% Volume Threshold, Medicare	
**Code**	**Code Description - Patient Language**	**UPP**	**PEAPP**	**Corporate**	**Hospital**	**HOPD as % of Lower-Cost Setting**	**Difference, HOPD vs. Lower-Cost Setting ($)**
**Cardiology**							
78452*	Myocardial SPECT imaging	73%	76%	73%	38%	330%	$996
93306*	Echocardiography, Doppler	61%	66%	56%	31%	303%	$397
93351*	Echocardiography, stress test	85%	86%	85%	54%	262%	$375
93880*	Extracranial arteries, bilateral study	87%	93%	84%	52%	143%	$81
93970*	Extremity veins, bilateral study	83%	89%	80%	40%	142%	$79
**Gastroenterology**							
43235	Esophagogastroduodenoscopy, diagnostic	31%	32%	27%	13%	167%	$394
43239	Esophagogastroduodenoscopy, biopsy	58%	60%	53%	24%	165%	$394
43248	Esophagogastroduodenoscopy, guide wire	62%	66%	58%	23%	162%	$394
43249	Esophagogastroduodenoscopy, balloon dilation	44%	48%	44%	19%	200%	$983
45378	Colonoscopy, diagnostic	51%	52%	45%	21%	161%	$398
45380	Colonoscopy, biopsy	64%	69%	60%	30%	163%	$513
45381	Colonoscopy, submucosal injection(s)	54%	55%	45%	23%	163%	$513
45384*	Colonoscopy, lesion removal with forceps	62%	73%	62%	34%	277%	$863
45385	Colonoscopy, snare technique lesion removal	66%	71%	62%	30%	160%	$513
45390	Colonoscopy, mucosal resection	39%	39%	26%	20%	179%	$1,329
**Orthopedics**							
22551	Arthrodesis, anterior interbody	6%	7%	5%	2%	135%	$3,683
23472	Arthroplasty, glenohumeral joint	2%	2%	1%	1%	124%	$3,772
27130	Total hip arthroplasty	6%	8%	5%	2%	131%	$3,308
27447	Total knee arthroplasty	8%	12%	7%	2%	134%	$3,498
29826	Shoulder arthroscopy, subacromial decompression	46%	46%	48%	23%	**	**
29827	Shoulder arthroscopy, rotator cuff repair	46%	46%	47%	24%	177%	$3,430
29881	Knee arthroscopy, meniscectomy	48%	54%	51%	25%	176%	$1,568
29888	Arthroscopic ACL repair	36%	36%	36%	22%	142%	$2,324

63047	Laminectomy, facetectomy and foraminotomy	7%	7%	6%	2%	176%	$3,430
64721	Neuroplasty and/or transposition	58%	52%	58%	33%	170%	$944
**Urology**							
50590	Lithotripsy, extracorporeal shock wave	23%	29%	28%	15%	177%	$1,699
52000*	Cystourethroscopy	89%	96%	94%	71%	305%	$491
52310*	Cystourethroscopy, removal of foreign body	76%	87%	84%	65%	656%	$1,773
52332	Cystourethroscopy, insertion of stent	11%	15%	14%	6%	195%	$1,699
52356	Cystourethroscopy, ureteroscopy/pyeloscopy, lithotripsy and stent	14%	23%	20%	7%	186%	$2,464
52442*	Cystourethroscopy, insertion of transprostatic implant	67%	82%	75%	32%	**	**
55700*	Prostate biopsy	76%	91%	84%	55%	861%	$1,831

Notes:

- Under the “Cost” heading, the percentages represent the HOPD payment as a percentage of, not in addition to, the payment for the lower-cost setting. The dollar amount is the delta between total payments for the HOPD and the lower-cost setting.
- *These codes use the office setting as the lower-cost SOC.
- **These codes are add-on codes with no separately payable facility fee.
- ***Note that the percentages included in these columns are probability results from our Medicare analysis; therefore, they do not contemplate the total volume of the service being provided (segmented by SOC), but rather the probability that each of the four affiliation models of specialty physicians performed the specific procedure in one of two lower cost settings (either the ASC or the office). The percent of total volume in Supplemental Table 3E provides a different set of metrics.

Supplementary Table S3E Summary Statistics by Procedure Code, Commercial

		Lower Cost Setting of Analysis	Volume, Lower Cost Setting (ASC or Office) vs. HOPD, 2022 Commercial Claims Volume*		Cost, HOPD Relative to Lower-Cost Setting (ASC or Office) Above 10% Volume Threshold, Commercial	
**Code**	**Code Description - Patient Language**	**Lower Cost Setting (Office vs. ASC)**	**Lower Cost Setting % Volume**	**HOPD % Volume**	**HOPD as % of Lower-Cost Setting**	**Difference, HOPD vs. Lower-Cost Setting ($)**
**Cardiology**						
78452	Myocardial SPECT imaging	Office	56%	39%	416%	$1,962
93306	Echocardiography, Doppler	Office	42%	35%	292%	$724
93351	Echocardiography, stress test	Office	66%	31%	251%	$662
93880	Extracranial arteries, bilateral study	Office	83%	14%	238%	$363
93970	Extremity veins, bilateral study	Office	73%	15%	253%	$357
**Gastroenterology**						
43235	Esophagogastroduodenoscopy, diagnostic	ASC	28%	42%	311%	$2,564
43239	Esophagogastroduodenoscopy, biopsy	ASC	51%	37%	289%	$2,515
43248	Esophagogastroduodenoscopy, guide wire	ASC	55%	39%	306%	$2,524
43249	Esophagogastroduodenoscopy, balloon dilation	ASC	40%	50%	250%	$2,907
45378	Colonoscopy, diagnostic	ASC	58%	32%	219%	$1,848
45380	Colonoscopy, biopsy	ASC	56%	36%	241%	$2,442
45381	Colonoscopy, submucosal injection(s)	ASC	47%	42%	303%	$3,122
45384	Colonoscopy, lesion removal with forceps	Office	57%	27%	561%	$3,439
45385	Colonoscopy, snare technique lesion removal	ASC	57%	36%	219%	$2,348
45390	Colonoscopy, mucosal resection	ASC	31%	64%	236%	$3,594
**Orthopedics**						
22551	Arthrodesis, anterior interbody	ASC	13%	54%	111%	$2,881
23472	Arthroplasty, glenohumeral joint	ASC	11%	69%	128%	$6,896
27130	Total hip arthroplasty	ASC	17%	65%	130%	$6,712
27447	Total knee arthroplasty	ASC	16%	68%	130%	$6,576

29826	Shoulder arthroscopy, subacromial decompression	ASC	53%	46%	178%	$5,528
29827	Shoulder arthroscopy, rotator cuff repair	ASC	51%	48%	180%	$8,242
29881	Knee arthroscopy, meniscectomy	ASC	53%	45%	193%	$4,070
29888	Arthroscopic ACL repair	ASC	52%	47%	188%	$9,565
63047	Laminectomy, facetectomy and foraminotomy	ASC	14%	41%	163%	$6,063
64721	Neuroplasty and/or transposition	ASC	49%	45%	197%	$3,127
**Urology**						
50590	Lithotripsy, extracorporeal shock wave	ASC	28%	67%	174%	$4,186
52000	Cystourethroscopy	Office	75%	13%	1032%	$3,224
52310	Cystourethroscopy, removal of foreign body	Office	70%	17%	1000%	$4,212
52332	Cystourethroscopy, insertion of stent	ASC	10%	56%	233%	$5,157
52356	Cystourethroscopy, ureteroscopy/pyeloscopy, lithotripsy and stent	ASC	16%	73%	228%	$6,178
52442	Cystourethroscopy, insertion of transprostatic implant	Office	35%	39%	800%	$10,203
55700	Prostate biopsy	Office	56%	27%	1346%	$4,982

Notes:

- Under the “Cost” heading, the percentages represent the HOPD payment as a percentage of, not in addition to, the payment for the lower-cost setting. The dollar amount is the delta between total payments for the HOPD and the lower-cost setting.
- *Note that the percentages included in these two rows represent the percent of total volume for the listed procedure in 2022, regardless of setting, that occurs in the setting that is specified. We used this commercial data for our service identification process, and then upon identifying codes that met our inclusion criteria, conducted our Medicare probability analysis.
- We chose to use commercial volume data for our code selection methodology because a) we wanted to be consistent in the codes we analyzed across Medicare and commercial, and b) given there are more limitations in the availability of the commercial data, we felt it was important to start there to ensure we had sufficient coverage of the codes we selected for analysis.

Supplementary Table S4: Distribution of patients and physicians by specialty and affiliation

Cardiology	Independent	Hospital	Corporate	PE
**Number of Physicians**	2,686	12,745	4,812	238
**Number of Patients**	887,846	3,800,083	1,778,733	96,529
**Gastroenterology**	**Independent**	**Hospital**	**Corporate**	**PE**
**Number of Physicians**	1,704	4,368	5,607	1,826
**Number of Patients**	309,323	421,618	756,871	304,983
**Orthopedics**	**Independent**	**Hospital**	**Corporate**	**PE**
**Number of Physicians**	3,139	7,321	10,638	938
**Number of Patients**	414,619	843,878	1,563,925	155,139
**Urology**	**Independent**	**Hospital**	**Corporate**	**PE**
**Number of Physicians**	1,266	3,394	2,498	843
**Number of Patients**	336,192	533,781	769,638	321,355

Supplemental Table S5: Logistic Regression results of Figure 3

	Cardiology		Gastroenterology		Orthopedics		Urology	
PEAPP affiliation	1.77	**	1.70	**	1.02	**	1.23	**
Corporate affiliation	1.30	**	1.33	*	0.90	*	0.99	*
Independent	1.53	**	1.53	*	0.98	*	0.71	*
PE dominated market	-0.25		0.11		0.05		-0.04	
Corporate dominated market	-0.12		-0.02		0.01		0.00	
Hospital dominated market	-0.39		-0.32		-0.08		-0.05	
Age	-0.01	*	-0.02	*	-0.03	*	0.02	*
Male	0.05	*	-0.04	*	0.20	*	0.31	*
Race_Asian	0.35		0.15		-0.08		0.19	
Race_Black	0.10		-0.07	*	-0.03	*	0.12	*
Race_His	0.26		0.02		-0.06		0.26	
Race_Other	0.11		0.08		0.00		0.04	
Death_year	-1.76	**	-1.51	**	-0.47	**	-0.74	**
HCC	-0.11	**	-0.32	**	-0.20	**	-0.08	**
Rural	-0.36		-0.38		-0.30		-0.28	
Medium household income	0.00		0.00		0.00		0.00	
Dual Medicaid Enrollment	-0.33		-0.49		-0.30		-0.22	
Entitlement_disability	-0.22		-0.36		-0.07		-0.04	
Entitlement_esrd	-0.30		-0.52		-0.29		0.10	
Entitlement_disability and esrd	0.21		0.40		0.39		-0.02	
Census_midwest	-0.66		-0.46		-0.13		-0.33	
Census_northest	0.24		-0.15		-0.19		-0.09	
Census_west	-0.12		0.10		0.07		0.06	
total number of Part D enrollment months	0.07		0.05		0.15		-0.05	

** P<0.01 (Wald-chi square test)
*P<0.05 (Wald-chi square test)
++ Overall chi-square test P<0.05
++ Psuedo R^2: 0.20
++ Analysis conducted in SAS Enterprise

Supplemental Table S6: Logistic Regression Regression results of Figure 4

	Cardiology		Gastroenterology		Orthopedics		Urology	
PEAPP affiliation	1.248	**	1.705	**	1.023	**	1.234	**
Corporate affiliation	1.299	**	1.335	*	0.902	*	0.995	*
Independent	1.531	**	1.533	*	0.981	*	0.713	*
PE dominated market	-0.243		0.113		0.047		-0.037	
Corporate dominated market	-0.118		-0.019		0.013		0.002	
Hospital dominated market	-0.390		-0.322		-0.076		-0.053	
Age	-0.009	*	-0.023	*	-0.029	*	0.020	*
Male	0.053	*	-0.039	*	0.202	*	0.312	*
Race_Asian	0.348		0.151		-0.079		0.193	
Race_Black	0.108		-0.061	*	-0.027	*	0.119	*
Race_His	0.264		0.025		-0.063		0.260	
Race_Other	0.110		0.076		-0.005		0.036	
Death_year	-1.718	**	-1.484	**	-0.477	**	-0.734	**
HCC	-0.107	**	-0.325	**	-0.199	**	-0.083	**
Rural	-0.365		-0.386		-0.304		-0.281	
Medium household income	0.000		0.000		0.000		0.000	
Dual Medicaid Enrollment	-0.367		-0.516		-0.291		-0.235	
Entitlement_disability	-0.210		-0.355		-0.067		-0.036	
Entitlement_esrd	-0.299		-0.520		-0.295		0.103	
Entitlement_disability and esrd	0.203		0.390		0.388		-0.028	
Census_midwest	-0.666		-0.461		-0.133		-0.329	
Census_northest	0.224		-0.155		-0.190		-0.088	
Census_west	-0.117		0.097		0.071		0.062	
total number of Part D enrollment months	0.012		0.008		-0.001		0.003	*

** P<0.01 (Wald-chi square test)
*P<0.05 (Wald-chi square test)
++ Overall chi-square test P<0.05
++ Psuedo R^2: 0.20
++ Analysis conducted in SAS Enterprise

Supplemental Table S7: Confidence Intervals of Results Presented in Figures 3 and 4

**CI Table for Figure 3**	**Corporate**		**Hospital**		**Independent**		**PE**	
**Procedure Code**		95% CI		95% CI		95% CI		95% CI
**Cardiology 93306**	55%	(53,56%)	31%	(30%,32%)	61%	(59%,63%)	66%	(65%,67%)
**Gastroenterology 45385**	62%	(60%,64%)	30%	(29%,31%)	66%	(64%,68%)	71%	(69%,73%)
**Orthopedics27447**	7%	(5%,9%)	2%	(1%,3%)	8%	(7%,9%)	12%	(11%,13%)
**Urology52000**	94%	(92%,96%)	71%	(69%,73%)	89%	(87%,91%)	96%	(95%,97%)
**CI Table for Figure 4**	**Corporate**		**Hospital**		**Independent**		**PE**	
**Specialty**		95% CI		95% CI		95% CI		95% CI
**Cardiology**	63%	(61%,65%)	34%	(33%,35%)	68%	(66%,70%)	72%	(70%,74)
**Gastroenterology**	54%	(52%,56%)	26%	(24%,28%)	59%	(57%,61%)	62%	(60%,64%)
**Orthopedics**	20%	(19%,21%)	9%	(8%,10%)	21%	(20%,22%)	21%	(20%,22%)
**Urology**	82%	(80%,84%)	98%	(97%,99%)	98%	(96%,100%)	96%	(95%,97%)
**Weighted**	63%	(61%,65%)	34%	(32%,36%)	59%	(57%,61%)	72%	(70%,74%)
